# *Candida albicans* at Host Barrier Sites: Pattern Recognition Receptors and Beyond

**DOI:** 10.3390/pathogens8010040

**Published:** 2019-03-25

**Authors:** Marc Swidergall

**Affiliations:** 1 Division of Infectious Diseases, Harbor-UCLA Medical Center, Torrance, CA 90502, USA; mswidergall@labiomed.org; 2 Institute for Infection and Immunity, Los Angeles Biomedical Research Institute at Harbor-UCLA Medical Center, Torrance, CA 90502, USA

**Keywords:** antifungal immunity, host–fungus interaction, mucosal tissue

## Abstract

Over the last decades, fungal infections have emerged as a growing threat to human health. Although the human body is at potential risk, various body sites host several commensal fungal species, including *Candida albicans*. In healthy individuals, *C. albicans* colonizes different mucosal surfaces without causing harm, while under diverse circumstances the fungus can proliferate and cause disease. In this context, the understanding of host–*C. albicans* interactions in health and during infection may lead to novel therapeutic approaches. Importantly, host cells express pattern recognition receptors (PRRs), which sense conserved fungal structures and orchestrate innate immune responses. Herein, important findings on the topic of the recognition of *C. albicans* at host barrier sites are discussed. This review briefly summarizes the importance and functions of myeloid PRRs, reviews the fungal recognition and biology of stromal cells, and highlights important *C. albicans* virulence attributes during site-specific proliferation and invasion.

## 1. Introduction

The human body is constantly threatened by the invasion of microorganisms, and has evolved systems of immune tolerance to commensal microbiota as well as immune defense mechanisms to eliminate pathogens on and in the body. The mammalian immune system is comprised of two branches: innate and adaptive immunity [1]. The innate immune system is the first line of host defense against pathogens. Depending on the anatomic site of microorganism invasion, various epithelial cell linings orchestrate the innate response to eliminate proliferating microorganisms. The adaptive immunity is characterized by specificityand is involved in the elimination of pathogens in the late phase of infection, as well as in the generation of immunological memory. 

The incidence of fungal infections is rising, and presents a serious threat to public health [2]. Nevertheless, this risk is relatively underappreciated by the press, the public, and funding agencies [3]. Some fungal infections are highly lethal and disproportionately affect vulnerable patients, such as neonates, as well as transplant and cancer patients. Approximately 15% of health-care associated infections are caused by fungi [4]. *Candida* spp. account for 70–90% of all invasive fungal infections, and are a frequent cause of hospital-acquired systemic infections in the United States with crude mortality rates of up to 50% [4]. Among the *Candida* spp., *C. albicans* and *C. glabrata* rank first and second in isolation frequency, respectively [5]. However, *C. albicans* is the most common fungal pathogen in most clinical settings [6]. The morphological flexibility of *C. albicans* plays a crucial role in several aspects of infection and host recognition [7], while the pathogenicity of non-albicans spp. lacking hyphal formation, such as *C. glabrata,* is independent of morphology. *C. albicans* can cause two major types of infections in humans: superficial infections, such as skin, oral, or vaginal candidiasis, and life-threatening systemic infections. While *C. albicans* resides on approximately 75% of mucosal surfaces in healthy individuals [8], its recognition is key to mount an antifungal response during fungal outgrowth in order to prevent disease. This review will provide current knowledge on the fungal recognition at barrier sites, with a focus on *C. albicans*.

## 2. Major “Classical” Fungal Pattern Recognition Receptors on Myeloid Cells

Pattern recognition receptors (PRRs) recognize a variety of pathogen-associated molecular patterns (PAMPs) expressed by invading microorganisms [9,10,11]. PAMP–PRR interactions on myeloid cells induce downstream mechanisms designed to eliminate the pathogen, including phagocytosis, respiratory burst, and immune mediator release [12].

Various C-type lectin-like receptors (CLRs), including Dectin-1, Dectin-2, and macrophage-inducible C-type lectin (Mincle), DC-Sign, and mannose receptor (MR), play major roles in antifungal immunity [13]. Dectin-1 recognizes exposed β-glucans in the cell wall of *Candida, Aspergillus*, and many other fungal species [14,15,16]. Ligand recognition activates the CARD9 pathway to trigger NF-κB activation, consequently inducing cytokine and chemokine production. In addition to inducting the secretion of cytokines and chemokines, Dectin-1 triggers phagocytosis and inflammasome activation in myeloid cells [17,18]. A homozygous polymorphism in Dectin-1 that eliminates expression on the cell surface confers increased susceptibility to recurrent vulvovaginal candidiasis (RVVC), but not oral mucosal infection [19]. The CLR Dectin-2, present on dendritic cells, macrophages, and neutrophils, recognizes α-mannan [20]. Dectin-2-deficient mice are more susceptible to systemic candidiasis [21]. Fungal–Dectin-2 interactions activate CARD9, resulting in subsequent NF-κB activation. Dectin-3 recognizes α-mannans on the surfaces of *C. albicans* hyphae and induces NF-κB [22]. The CLR Mincle induces phagocytosis, fungal killing, and induction of TNF, IL-6, CXCL2, and KC in macrophages after fungal recognition [23].

Toll-like receptors (TLRs) are evolutionarily conserved transmembrane proteins that sense extracellular and intracellular pathogens in endosomes and lysosomes. TLR signaling activates either MyD88-dependent or TRIF-dependent pathways [24]. Various fungal species, as well as β-glucans, induce TLR2 or TLR2/TLR6-mediated cytokine responses [25]. TLR2 senses phospholipidomannan on the fungal surface [26]. Furthermore, TLR2 is able to directly bind chitin to trigger inflammation, dependent on the oligomer size [27]. On note, TLR2 is recruited to zymosan β-glucan-containing phagosomes and induces caspase-3/8-mediated apoptosis in monocytes in response to *C. albicans*, suggesting synergy between CLRs and TLRs [28,29,30]. Strikingly, TLR2 knockout mice are more resistant to disseminated *Candida* infection due to a decrease in the CD4^+^CD25^+^ regulatory T (Treg) cell population [31]. TLR4 senses *O*-linked mannosyl chains in the cell wall of *C. albicans* [32]. TLR4 knockout mice are more susceptible to systemic *C. albicans* infection due to reduced chemokine responses and impaired neutrophil recruitment. However, cytokine responses are unaffected in TLR4 knockout macrophages [33]. Secreted *C. albicans* proteins are also able to bind and activate TLRs. A small secreted cysteine-rich protein secreted elicitor 1 (Sel1) can be sensed by both TLR2 and TLR4, leading to the activation of IKK/NF-κB and MAPK/AP-1 to trigger cytokine and TNFα production [34]. The intracellular receptor TLR9 recognizes unmethylated CpG DNA and is activated by fungal DNA to induce cytokine production in dendritic cells as well as phagosomal TLR9 recruitment in macrophages [35,36].

NOD-like receptors (NLRs) generally interact with apoptosis-associated speck-like protein containing a CARD (ASC) and procaspase-1 forming the inflammasome. In humans, polymorphisms in NLRs increase *C. albicans* colonization of the gut and NLRP3 polymorphism predisposes patients to RVVC [37]. In mice, macrophage-derived Nlrp3 recognizes *C. albicans* hyphae, and deficiency of ASC, NLRP3, and IL-1R1 leads to increased dissemination of mucosal *C. albicans* infection and mortality in vivo [38]. Additionally, stromal-cell-derived NLRC4 is critical for mediating immunity against oral candidiasis [39]. 

RIG-I-like (RLR) receptors are DEAD box helicases and cytoplasmic pathogen recognition receptors that recognize and bind to nonself RNA to trigger innate immunity [40]. In macrophages, the RLR MDA5 is specifically induced by *C. albicans* hyphae, and patients suffering from chronic mucocutaneous candidiasis (CMC) express lower levels of MDA5 than healthy controls [41]. Additionally, splenocytes from Mda5 knockout mice and human peripheral blood mononuclear cells (PBMCs) with different *IFIH1* genotypes display an altered cytokine response to *C. albicans*.

In addition to direct PRR interactions with fungal components myeloid receptor signaling is activated by host proteins bound to the fungal surface, including complement and antibodies [42]. Complement consists of approximately 30 fluid-phase and membrane-bound proteins that cooperate to form the cascade [43]. Following infection three different complement activation pathways commence rapidly to activate complement component 3 (C3) [44,45]. Opsonization with iC3b, a C3 fragment, is critical for fungal phagocytosis [46]. C3-deficient mice are more susceptible to infection with *C. albicans* due to impaired fungal clearance without affecting inflammatory responses [47]. The pentraxin family members PTX3 and serum amyloid P component (SAP) bind mannose-binding lectin (MBL) to trigger C1q recruitment resulting in enhanced *C. albicans* opsonophagocytosis by polymorphonuclear neutrophils (PMNs) [48]. In humans, polymorphisms in PTX3 are associated with the risk of *Candida* infection [49]. Elimination of immunoglobulin G (IgG)-opsonized *C. albicans* by neutrophils strictly depends on the Fcγ receptors, protein kinase C (PKC), and intracellular ROS accumulation, while non-opsonized *C. albicans* are recognized via complement receptor 3 (CR3; CD11b/CD18) inducing Syk and PI3K activation to clear phagocytosed *C. albicans* independent of ROS production [50]. Several PRRs, including Dectin-2, Dectin-3, and Mincle, associate with the FcγR signaling chain, which contains an immunoreceptor tyrosine-based activation motif (ITAM) to induce downstream signaling via Syk [22,51].

Cross-talk between myeloid PRRs, and within the corresponding innate immune pathways, enables the myeloid innate immune system to orchestrate immediate local and global responses [52,53]. Depending on different combinations of PAMPs, together with the order and dosage of sequential challenges, PRRs coordinate and amplify multiple pathways that are involved in antifungal responses and the regulation of cytokine synergy [54,55]. Along these lines, ITAM-coupled receptors cross-regulate signaling from heterologous receptors to fine-tune specific activation [56,57]. By contrast, certain cell types, such as natural killer (NK) cells, utilize specific PRRs to induce and amplify antifungal responses [58]. Thus, the broad repertoire of various PRRs on different myeloid cells ensures immediate recognition and antifungal mechanism during infection.

## 3. Barrier Sites in the Human Body

The skin and mucosa define the frontier between the internal milieu and the external nonsterile environment [59]. These barriers are under intense scrutiny by the immune system and the maintenance of these barriers is paramount. Invading microorganisms and their products, such as secondary metabolites, remain a constant threat. Other cellular barriers, such as the blood–brain barrier (BBB), characterize protected distinct niches within the host. In addition to their physiological roles, these host barriers provide immune defense mechanisms against fungal infection, such as *C. albicans* [60,61].

### 3.1. Skin Immunity against C. albicans

The skin surface is a unique niche, harboring a distinct community of bacteria and fungi [62,63]. In chronic mucocutaneous candidiasis (CMC) patients, *C. albicans* causes cutaneous lesions with the thickening, hyperkeratosis, and erythema of the skin [64,65]. In the skin, fungi interact primarily with keratinocytes, dendritic cells (DCs), and macrophages, as well as with myeloid cells that are recruited to the skin under inflammatory conditions [66,67]. The innate immune system and its effectors play a key role against cutaneous infections [68]. Fungal invasion of keratinocytes activates the expression of host defense peptides (HDPs), antimicrobial molecules, which inhibit fungal proliferation [69,70,71]. In addition to producing HDPs, keratinocytes secrete chemokines and cytokines in response to invading *C. albicans* [72]. TLR2 expression and activation in fibroblasts is required but not sufficient for protection against *C. albicans* invasion in a CD4^+^ T cell-supplemented human skin model [73]. Furthermore, melanocytes play a key role in the innate immune response of the skin by recognizing *C. albicans* via TLR4, resulting in increased melanin content and inhibition of infection [74]. Melanization produces intermediate toxic compounds which exhibit strong antimicrobial activities. Additionally, melanin—the end-product—may have the capacity to trap, inhibit, and even kill invading pathogens [75]. Due to their location within stratified epithelia, Langerhans cells (LCs) are part of the first line of defense against pathogens present in the environment. Non-activated LCs are constantly migrating to the lymph nodes to present self-antigen and establish immune tolerance in homeostatic conditions [76]. On LCs Dectin-1 recognizes *C. albicans* yeast, resulting in an IL-6-dependent Th17 response [77]. Recognition of *C. albicans* filaments by CD103^+^ dermal DCs (dDCs) through TLR-2 induces Th1 cell differentiation in the secondary lymphoid organs. Strikingly, these Th1 cells provide protection against a secondary systemic infection but not a secondary skin infection [77]. Additional recognition of invading fungi is provided by skin sensory neurons via the neuropeptide calcitonin-related gene product to direct IL-23–dependent γδ T cell IL-17 release, an integral cytokine of antifungal barrier immunity [78,79,80,81]. 

### 3.2. Oral Immunity in Response to C. albicans Recognition

A multitude of antigens derived from food and the resident bacterial as well as fungal community continuously interact with oral epithelial cells. The oral cavity hosts up to 100 different fungal species [82], and the epithelial barrier is able to discriminate between commensal and pathogenic fungal forms [83]. Due to local or systemic immunosuppression, commensal fungi are able to proliferate resulting in oral disease. The most prevalent fungal-related oral disease is oropharyngeal candidiasis (OPC) which is caused mainly by *C. albicans*. Predisposing factors for oral *C. albicans* outgrowth include but are not limited to use of dentures, corticosteroid inhalers, cigarettes, broad-spectrum antibiotics, and immunosuppressive and chemotherapeutic agents [84]. Patients with CMC are susceptible to debilitating, persistent, and refractory mucosal infections [85]. Mutations in *STAT1*, IL-23 and IL-12 receptors, *IL-17RA*, and *IL-17F* all lead to CMC [86]. Furthermore, patients with autosomal recessive autoimmune polyendocrinopathy candidiasis ectodermal dystrophy (APECED) have mutations in the *autoimmune regulator* (*AIRE*) gene [87], resulting in a loss-of-function phenotype that causes the production of neutralizing autoantibodies against important cytokines with antifungal properties such as IL-17E, IL-17F, and IL-22 [88]. Autoantibodies against IL-22 and IL-17F are more common in APECED patients with CMC than in those without CMC, suggesting that type Th17 cytokines are central in human epithelial immunity against *C. albicans* infection [89,90]. However, recent experimental data have shown that autoantibodies against murine IL-17A do not increase susceptibility to OPC, while anti-IL-22 antibodies derived from patients increase the oral fungal burden [91].

*C. albicans* proliferation and the initiation of hyphal growth are required to induce a strong antifungal response in the oral cavity. While “classical” myeloid PRRs, such as TLR2 and Dectin-1, are expressed by human oral epithelial cells [92,93], these receptors are dispensable for the host defense against OPC in mice [94,95]. In human oral epithelial cells, Dectin-1 induces NF-κB signaling in response to proliferating *C. albicans* [93]. Furthermore, exposed β-glucan on the *C. albicans* surface is recognized by epithelial ephrin type A 2 receptor (EphA2) [93]. Fungal outgrowth in the oral cavity induces strong receptor activation and consequently downstream signaling through signal transducer and activator of transcription 3 (STAT3) and mitogen-activated protein kinases (MAPKs), which activate a distinct profile of chemokines, cytokines, and host defense peptides. During acute OPC, EphA2 knockout mice have a diminished antifungal cytokine and chemokine response in the tongue, resulting in higher oral fungal burden and a more severe disease [93]. Importantly, β-glucan (zymosan) or heat-inactivated *C. albicans* induce transient EphA2 signaling indicating that secondary fungal and/or host responses are required to mount a full antifungal response by oral epithelial cells. Therefore, this secondary messenger response might explain why the ingestion of fungal β-glucans does not induce an inflammatory response in the oral cavity. 

*C. albicans* invades the epithelial cell lining of the oropharynx via distinct mechanisms. During fungal-induced endocytosis, *C*. *albicans* hyphae express invasins, which activate E-cadherin, the epidermal growth factor receptor (EGFR), and HER2 on the epithelial cell surface, resulting in internalization of the fungus [96,97]. During active penetration, *C. albicans* hyphae push their way into the epithelial cell [98]. Invasion of the oral epithelial cell lining results in tissue damage that is mainly caused by Candidalysin, a small toxin secreted by hyphal cells [99,100]. The damage of epithelial cells leads to IL-1 release, resulting in granulopoiesis and the expansion of innate CD4^+^ αβ T cell receptor (TCRαβ)^+^ cells [94,101]. TCRαβ^+^ cells, γδ T cells, and type-3 innate lymphoid cells (ILC3s) serve as IL-17 innate sources during acute OPC to amplify mucosal antifungal responses [102,103]. It is thought that the *C. albicans* morphology determines its pathogenicity in the oral cavity. However, a recent study shows that *C. albicans* strain variation modulates disease outcome in the oral cavity [104,105]. At the onset of oral infection the attenuation of inflammatory responses with a persistent/commensal *C. albicans* isolate is independent of Tregs and the immunoregulatory cytokine IL-10, and consequently independent of immunosuppression [104]. Strikingly, both pathogenic and commensal *C. albicans* strains induce conserved IL-17 signaling responses [105]. Importantly, *C. albicans*-induced epithelial damage correlates with a strong inflammatory response and rapid fungal clearance, while low epithelial cell damage is associated with oral colonization and fungal persistence [105]. However, lack of the damaging toxin Candidalysin does not abolish fungal clearance or *C. albicans* persistence in the immunocompetent host [94] suggesting that besides epithelial damage, other fungal factors, as well as host factors, contribute to pathogenicity during OPC and to fungal tolerance in homeostatic conditions.

### 3.3. C. albicans Recognition and Immunopathology during Vulvovaginal Candidiasis

Vulvovaginal candidiasis (VVC) is a common mucosal infection, caused primarily by *C. albicans.* Worldwide, recurrent VVC affects about 138 million women annually (range 103–172 million), with 372 million women affected by recurrent episodes over their lifetime [106]. VVC is characterized by itching, burning, pain, redness of the vulva and vaginal mucosa, and discharge [107]. Neutrophils play an essential role during VVC immunopathology. In a mouse model of VVC depletion of these cells results in similar fungal burden but decreased histological evidence of vaginal inflammation [108] suggesting that vaginitis symptoms are associated with PMN recruitment rather than fungal burden. Importantly, the yeast-to-hyphae transition is crucial to induce vaginal neutrophil immunopathogenesis. *C. albicans* recognition through epithelial TLR4 and specific intercellular adhesion molecule-3-grabbing nonintegrin-related 1 (SIGNR1) induces the expression of the alarmins S100A8/A9. These potent chemotactic factors recruit neutrophils to the site of infection [109]. Neutrophil recruitment is further amplified by Candidalysin-dependent epithelial damage [110]. Epithelial damage stimulates proinflammatory cytokine production, and consequently neutrophil recruitment [110]. In addition to innate phagocyte recruitment, neutrophil anergy contributes to chronic immunopathological state [111]. Vaginal heparan sulfate blocks the recognition of *C. albicans* Pra1 by neutrophils [112] consequently diminishing neutrophil activity and leading to PMN anergy [113].

### 3.4. Intestinal Immunity to C. albicans

The lower gastrointestinal (GI) fungal community consists of *Candida, Saccharomyces, Aspergillus, Cryptococcus, Malassezia, Cladosporium, Galactomyces,* and *Trichosporon* spp. [113]. Fungal dysbiosis, the alteration of the mycobiotic composition, results in altered local and systemic immunity through mechanisms that include the modulation of the cytokine milieu, the activation of different cell types and subsets, and the release of metabolites [114]. Intestinal epithelial cells, the mucus layer, HDPs, and IgA are crucial to maintain intestinal immunity [115,116]. IL-17/22-producing ILC3s and Th17 cells regulate defense peptide and IgA expression [117,118]. In humans, intestinal inflammation expands *C. albicans*-specific and cross-reactive Th17 cells [119]. In otherwise immunocompetent mice, *C. albicans* GI colonization is independent of Th17 immunity [120]. In patients with Crohn’s disease, the presence of *C. albicans* and *C. parapsilosis* is increased in the inflamed mucosa [121]. In mice, fungal recognition by CX3CR1-expressing mononuclear phagocytes predisposes intestinal inflammation [122]. Furthermore, in Crohn’s disease patients, a missense mutation in the gene encoding CX3CR1 is associated with impaired antifungal responses. *C. albicans*-driven IL-9 induction activates TGF-β in stromal mast cells (MCs) via the indoleamine 2,3-dioxygenase (IDO) enzyme, leading to intestinal immune tolerance, barrier permeability, inflammation, and dissemination [123]. 

In a mouse model of GI dissemination *C. albicans* translocation requires a combination of bacterial dysbiosis, fungal colonization, neutropenia, and intestinal barrier dysfunction [124]. The hyphae-associated *C. albicans* invasin Als3 facilitates intestinal cell invasion [125]. Additionally, *C. albicans*-mediated necrotic epithelial damage mediates translocation through the intestinal barrier via a transcellular route [126]. 

### 3.5. Endothelial Recognition of and Immunity against C. albicans

The dissemination of fungal organisms starts with their entry into the bloodstream. *C. albicans* bloodstream infections arise from either transmigration of the fungus through the mucosal barrier of the GI tract or from biofilm formation on prosthetic material, such as intravenous catheters [127]. Kidneys are a primary target organ of *C. albicans*, and fungal invasion leads to loss of renal function and death [128]. Exit from the circulation occurs by fungal adhesion to, and then penetration into, the endothelial lining of blood vessel. The interaction of *C. albicans* with the endothelial lining and blood vessel invasion involve a complex series of processes [129]. *C. albicans* hyphae bind to N-cadherin on the surface of endothelial cell, thus triggering endocytosis of the organism [130,131,132]. However, the role of N-cadherin and receptor-mediated endocytosis to induc antifungal responses in endothelial cells remains unknown. Endothelial cells express many “classical” PRRs, including TLR2, TLR3, TLR4, and TLR9 [133]. On endothelial cells, TLR3, NF-κB, and p38 MAPK pathways trigger proinflammatory gene expression in response to *C. albicans* invasion [134]. *In vitro* endothelial cells respond to infection with *C. albicans* by secreting TNFα, which stimulates CXCL8/IL-8 and E-selectin expression by an autocrine mechanism [135,136]. Depletion of MyD88 and IL-1R-associated kinase-1 (IRAK) diminishes *C. albicans*-induced NF-κB activation [134]. Furthermore, fungal-induced CXCL8/IL-8 expression is mediated by TLR3, rather than TLR2 and TLR4. Upregulation of vascular cell adhesion molecule 1 (VCAM-1) depends on membrane-bound TNFα and IL-1α/β, while intercellular adhesion molecule 1 (ICAM-1) expression is induced by a different mechanism. *C. albicans*-mediated chemokine expression critically depends on the NF-κB signaling pathway [135,136]. Thus, the production of leukocyte adhesion molecules and proinflammatory mediators by endothelial cells, is a crucial step to circumvent *C. albicans* dissemination after fungal recognition. 

### 3.6. The Blood–Brain Barrier and C. albicans

The blood–brain barrier (BBB) maintains the homeostasis of the central nervous system (CNS) microenvironment by restricting the access of macromolecules, cells, and pathogens. In forming the BBB, brain endothelial cells limit the diffusion of large or hydrophilic molecules into the central nervous system, while allowing the diffusion of small hydrophobic molecules [137]. Several studies in mice and humans have shown that *C. albicans* is able to cross the BBB [138,139,140,141,142].

Binding of *C. albicans* to human microvascular endothelial cells induces pseudopod-like structures and intracellular vacuole-like structures retaining *C. albicans* [143]. Fungal internalization occurs when the specific brain endothelial receptor heat shock protein 90kDa beta member 1 (gp96) binds to hyphal invasins [144]. In addition to invading the BBB by fungal-induced endocytosis, several microorganisms, including fungi, utilize a Trojan horse mechanism to invade the brain [145]. In this mechanism, phagocytes such as macrophages and monocytes phagocytose the fungus and carry it across the BBB and into the brain. Whether *C. albicans* utilizes a Trojan horse-like mechanism to invade the brain remains unclear. Microglia are the most frequent innate immune cells in the central nervous system. Upon *C. albicans* brain infection, microglia produce CXCL1 and CXCL2 in a CARD9-dependent manner recruiting CXCR2^+^ neutrophils, which are required to control *C. albicans* proliferation in the brain [138,146].

## 4. Conclusions

The field of antifungal barrier immunity has rapidly advanced in the past decade, marked by the dissection of barrier-specific immune responses in conjunction with human clinical data combined with knowledge from animal models of infection. The precise recognition of commensal and pathogenic *C. albicans* forms and the subsequent initiation of an antifungal network prevents fungal outgrowth in healthy individuals. The identification of novel host receptors, innate immune pathways, and fungal virulence attributes not only have important consequences for our understanding of the immune response to *C. albicans*, but they also open new avenues for future research. Moreover, several crucial questions related to barrier antifungal immunity remain unanswered. For example, which factors contribute to mucosal fungal tolerance in homeostatic conditions, and are mucosal persistent/commensal *C. albicans* isolates less virulent during systemic candidiasis? Additional fungal recognition receptors, antifungal pathway synergy, and the mechanism to initiate antifungal responses remain to be discovered, both in animal models and in human genetic studies. 
